# Design and preliminary report of a randomized phase IIb clinical trial of multitargeted recombinant adenovirus 5 vaccines against CEA, MUC1, and brachyury (Tri-Ad5) and the IL-15 receptor superagonist nogapendekin alfa inbakicept in Lynch syndrome (TRIAD5-Plus): the first cross-network trial of the Cancer Prevention Clinical Trials Network (CP-CTNet)

**DOI:** 10.3389/fimmu.2026.1809281

**Published:** 2026-05-19

**Authors:** Ajay Bansal, Elena M. Stoffel, Ramona Lim, Peter P. Stanich, Swati G. Patel, Carol A. Burke, Michael J. Hall, Aparajita Singh, Lisa A. Boardman, Niloy Jewel Samadder, Veroushka Ballestar, Charles Muller, Aaron James Scott, Kelly A. Benante, Yanfei Xu, Jens Eickhoff, Meredith Hyun, Jen Zaborek, Lanni Aquila, Asad Umar, Eva Szabo, Gary Della’Zanna, Ellen Richmond, Lisa Bengtson, Jeffrey Schlom, Renee N. Donahue, Nicole J. Toney, Lucas A. Horn, Claudia Palena, James L. Gulley, Sohini Samaddar, Dennis F. Moore, Parth Sharma, Rashna Madan, Karen E. Hurley, Patrick Soon-Shiong, Elizabeth Gabitzsch, Lennie Sender, Dean Brenner, Scott Schuetze, Zora Djuric, Lisa M. Barroilhet, Patricia Thompson, Julie Bauman, Seema A. Khan, Eduardo Vilar

**Affiliations:** 1 Department of Gastroenterology, University of Kansas Medical Center, and University of Kansas Cancer Center, Kansas City, KS, United States; 2 Department of Internal Medicine, University of Michigan, Ann Arbor, MI, United States; 3 Department of Gastroenterology, Dana-Farber Cancer Institute, Boston, MA, United States; 4 Division of Gastroenterology, Hepatology and Nutrition, The Ohio State University Wexner Medical Center, Columbus, OH, United States; 5 Department of Medicine-Gastroenterology, University of Colorado, Aurora, CO, United States; 6 Department of Gastroenterology, Hepatology and Nutrition, Cleveland Clinic, Cleveland, OH, United States; 7 Department of Clinical Genetics, Fox Chase Cancer Center, Philadelphia, PA, United States; 8 Division of Gastroenterology, University of California, San Francisco, San Francisco, CA, United States; 9 Division of Gastroenterology and Hepatology, College of Medicine, Mayo Clinic, Rochester, MN, United States; 10 Division of Gastroenterology and Hepatology, Department of Medicine, Mayo Clinic, Phoenix, AZ, United States; 11 Department of Medicine, University of Puerto Rico Comprehensive Cancer Center, San Juan, Puerto Rico; 12 Division of Gastroenterology and Hepatology, Northwestern University, Chicago, IL, United States; 13 Division of Hematology and Oncology, University of Arizona Cancer Center, Tucson, AZ, United States; 14 Robert H. Lurie Comprehensive Cancer Center, Northwestern University Feinberg School of Medicine, Chicago, IL, United States; 15 University of Wisconsin Carbone Cancer Center, University of Wisconsin-Madison, Madison, WI, United States; 16 Frontier Science Foundation, Amherst, NY, United States; 17 Division of Cancer Prevention, National Cancer Institute, National Institutes of Health, Bethesda, MD, United States; 18 Center for Immuno-Oncology, Center for Cancer Research, National Cancer Institute, National Institutes of Health, Bethesda, MD, United States; 19 Department of Internal Medicine, University of Kansas Medical Center, Kansas City, KS, United States; 20 Cancer Center of Kansas, Wichita, KS, United States; 21 Department of Hematology and Oncology, Virginia Commonwealth University Medical Center, Richmond, VA, United States; 22 Department of Pathology and Laboratory Medicine, University of Kansas Medical Center, Kansas City, KS, United States; 23 Neurological Institute, Department of Psychiatry & Psychology, Center for Adult Behavioral Health, Cleveland Clinic, Cleveland, OH, United States; 24 ImmunityBio, Culver City, CA, United States; 25 Division of Gynecologic Oncology, University of Wisconsin, Madison, WI, United States; 26 Department of Physiology, University of Arizona Cancer Center, Tucson, AZ, United States; 27 Department of Surgery, Northwestern University Feinberg School of Medicine, Chicago, IL, United States; 28 Department of Clinical Cancer Prevention, The University of Texas MD Anderson Cancer Center, Houston, TX, United States

**Keywords:** cancer interception, cancer prevention, colorectal cancer, IL-15 receptor superagonist, immunity-based vaccination, Lynch syndrome, tumor-associated antigens

## Abstract

**Introduction:**

Lynch syndrome (LS) is a hereditary cancer syndrome that increases risk for colorectal and other cancers. We hypothesize that vaccines against tumor-associated antigens CEA, MUC1, and brachyury, simultaneously delivered in an adenovirus serotype-5 vector (Tri-Ad5) combined with the immune-enhancing IL-15 receptor superagonist nogapendekin-alfa-inbakicept (NAI) will reduce the incidence of colorectal neoplasms in LS carriers.

**Methods:**

In this ongoing phase IIB double-blind placebo-controlled trial, two open-label safety phases (SPs) assessed safety of Tri-Ad5 alone (SP1, n=10) and with added NAI (SP2, n=10). A randomized controlled trial (RCT) follows the SPs. After baseline colonoscopy, vaccine dosing occurs at weeks 0, 4 and 8; Tri-Ad5 alone in SP1 and Tri-Ad5 plus NAI in SP2, with identical boosters at Week 52. The RCT phase participants (n=138) were randomized to receive Tri-Ad5 plus NAI or placebo. All participants complete colonoscopy at Weeks 52 and 104 for assessment of the primary endpoint: cumulative colorectal neoplasm incidence. Secondary endpoints include safety, tolerability, and immunogenicity.

**Results:**

All 20 SP participants (median age 57.5 (range 42–75), 70% female, 15% minority) received all prime and booster series. SP1 participants reported 139 adverse events (AEs) and SP2 participants reported 178. AEs were predominantly grade 1; no treatment-related serious adverse events (SAEs) occurred. The most common treatment-related AEs were reactogenic events including grade 3 rash (without skin necrosis or breakdown) at NAI injection site (100% of SP2 participants). The RCT phase (n=138) recently completed accrual.

**Discussion:**

In LS carriers without active cancer, the combination of Tri-Ad5 + NAI was well-tolerated; the RCT phase is ongoing.

**Clinical Trial Registration:**

https://clinicaltrials.gov/study/, identifier NCT05419011.

## 1 Introduction

Current options for cancer interception in Lynch syndrome (LS), the most common inherited gastrointestinal cancer syndrome, are limited (1–3). LS occurs due to germline mutations in one of four DNA mismatch repair (MMR) genes, including *MLH1, MSH2, MSH6, PMS2*, or deletions affecting the 3′ region of *EPCAM* (4). A defective MMR system leads to the accumulation of hundreds of somatic mutations that drive carcinogenesis in LS carriers, although the exact sequence of mutational events might be individual-specific (5, 6).

LS contributes to 2–3% of all colorectal cancers (CRC) with a cumulative risk of development of CRC in LS carriers estimated to be as high as 82% (7, 8). Additionally, LS predisposes to the development of extracolonic malignancies, including endometrial, ovarian, gastric, small intestinal, urinary tract, pancreaticobiliary, brain, and skin tumors (8, 9). LS carriers are subjected to multiple screening procedures annually/bi-annually, and many undergo risk-reducing surgeries that significantly impact their quality of life. Therefore, novel cancer interception strategies that could reduce the incidence of multiple cancer types in LS are urgently needed.

Pharmacologic chemoprevention trials have led to the recommendation of high-dose aspirin (600 mg/day) for prevention of colorectal cancers, but not extracolonic malignancies (10, 11). However, uptake has been very limited, in part because of prescribers’ and patients’ concerns around potential side effects (12–14). Recently, the Cancer Prevention Project 3 (CaPP3) compared three different dose levels of aspirin (100 mg, 300 mg and 600 mg given daily) for cancer prevention in LS (15) with results that await publication. Given the extensive immune activation observed in cytotoxic CD8^+^ T cells in LS neoplasms, immune interception is an appealing approach (16, 17). Vaccination strategies may prime T cells to recognize cancer antigens on LS adenomas and early-stage tumors to shift immune surveillance from equilibrium to elimination at the pre-malignant stage (18).

Our trial employs a vaccination strategy that addresses common reasons for vaccine failure by targeting multiple antigens, using a proven adenoviral delivery system, and studying an immune-competent high-risk population without active cancer. We utilized a novel trivalent vaccine combination also known as ETBX-081 targeting three specific tumor-associated antigens (TAAs): mucin1 (MUC1, ETBX-051), carcinoembryonic antigen (CEA, ETBX-011), and brachyury (ETBX-061) (19, 20) (all known to be altered in multiple tumor types) (21–29). In these individual vaccines, transgenes reflecting T-cell enhancer agonist epitopes of CEA, brachyury, and MUC1 (25, 30–32) including those of the C-terminus of MUC1 (which has been shown to be an oncogene) (33), have been encoded in proprietary third-generation adenoviral gene delivery vectors that are designed to circumvent Ad5 pre-existing immunity with unique deletions of the viral DNA polymerase and the pre-terminal protein region (Ad5 [E1-, E2b-] (34–36). This vaccine is being used in combination with nogapendekin alfa inbakicept (NAI), which is a synthetic modified cytokine fusion protein that is an IL-15 receptor superagonist, to promote recruitment of natural killer (NK) cells and CD8^+^ T cells (37–40). NK cells are increasingly being recognized to regulate host responses to cancer and promote anti-tumor immunity (41–44). In combination with tumor vaccines, NAI has proved to boost immune responses further, improved survival, and increased the ratio of CD8^+^ T cells to regulatory T cells in preclinical models (40, 45). A phase 1 clinical trial of the novel trivalent combination vaccine in patients with advanced colon cancer has demonstrated a well-tolerable safety profile and promising immunogenicity (20). Moreover, trials in healthy volunteers confirmed both the safety and immune-activating capacity of NAI (38), thus providing the rationale for using it as an immune stimulant with the Tri-Ad5 vaccine. The current trial marks the first time the three vaccines and NAI are being tested in a cancer prevention setting. In this paper, we primarily describe the overall study design and present preliminary results of the safety phases.

## 2 Methods

### 2.1 Trial planning and execution

The trial is supported by the Cancer Prevention—Clinical Trials Network (CP-CTNet), a grant-funded network from the National Cancer Institute’s (NCI) Division of Cancer Prevention (DCP) that performs early-phase cancer prevention clinical trials. This trial represents the first cross-network trial performed by all five grant recipients and their affiliated organizations, at a total of 16 study sites. The geographic distribution of these sites has allowed participants nationwide to access the trial. The CP-CTNet sites are supported by the Data Management, Auditing, and Statistical Center (DMASC), which provides multidisciplinary expertise in information technology, clinical research informatics, data management and reporting, clinical trials auditing, clinical trials methodology and biostatistics, as well as operations management for CP-CTNet clinical trials.

### 2.2 Study design

**Figure 1:**
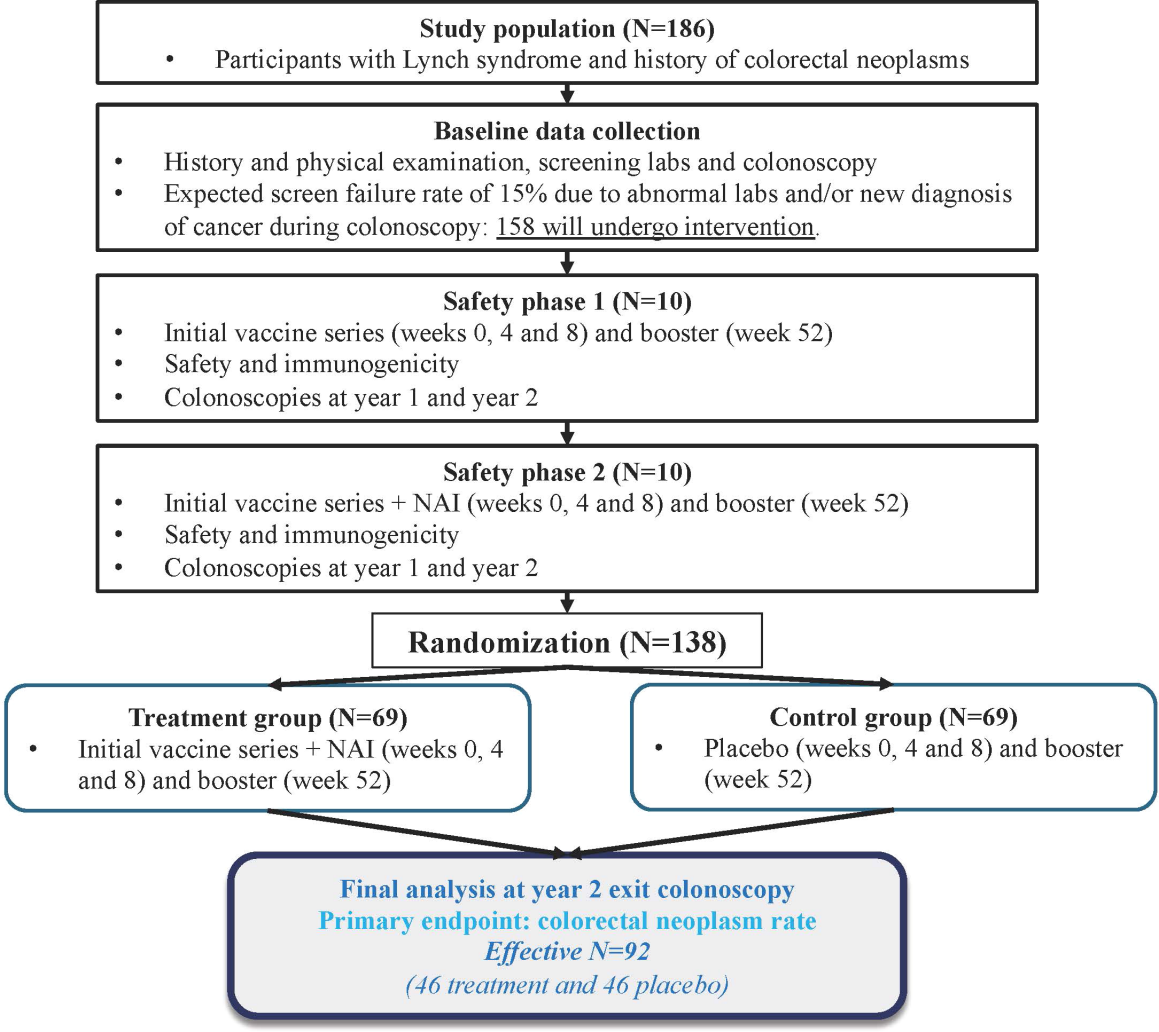
Study schema.

This is a randomized phase IIb, double-blind, placebo-controlled multicenter trial to evaluate the safety and efficacy of the combination of three adenovirus-5 (Tri-Ad5) vaccines and IL-15 receptor superagonist NAI in reducing colorectal neoplasm incidence in individuals with LS (**Figure 1**). This study has been designed to enroll 158 eligible participants across 16 institutions, accounting for a cumulative screen failure rate of 33.2%.

Two open-label safety phases were included prior to the randomized controlled trial (RCT) phase to assess the safety profiles of the Tri-Ad5 vaccines alone (Safety Phase 1, SP1) and after the addition of NAI (Safety Phase 2, SP2). Although previous experience with the vaccines and NAI demonstrated safety and good tolerability, SP1 and SP2 were included since potential participants to be enrolled were likely more immunocompetent than those with advanced cancer enrolled in prior studies. SP1 enrolled 10 participants, who received the three vaccines at Weeks 0, 4, and 8 (prime series), at a dose of 5x10^11^ viral particles (VP) per injection, followed by a booster at the same dose at Week 52. In SP2, 10 participants received the same doses and schedules of the three adenoviral vaccines as well as concurrent NAI at Weeks 0, 4, and 8, at a dose of 15 μg/kg (prime series), followed by a booster dose of NAI with vaccines at Week 52. The vaccines were injected subcutaneously at separate sites in two lower limbs (two vaccines in one thigh, at least 5 cm apart, and one vaccine in the other thigh) and the sites were rotated during subsequent series. NAI was injected subcutaneously into one of the four quadrants on the abdominal wall with rotation between quadrants during subsequent series. During SP1 and SP2, safety and toxicity were evaluated continually with a planned evaluation by the Data Safety and Monitoring Board (DSMB) before proceeding to the double-blind randomization phase.

The RCT phase was designed to enroll 138 participants randomly assigned in a 1:1 ratio to receive either the same regimen as SP2 or saline placebos. All participants received four separate injections (active or placebo): three injections in two limbs and one injection in the abdominal wall.

Given the already known frequency of skin reactions with NAI administration from the SP2 (**see Results below**), dose modification for NAI/placebo to a dose of 10 μg/kg per injection was allowed at Weeks 4, 8, and 52 during the RCT phase for dermatologic side effects that were considered unacceptable to the participant.

### 2.3 Eligibility criteria

**Table 1** lists the key inclusion and exclusion criteria. A major eligibility criterion is a history of colorectal neoplasms because vaccines are expected to enhance preexisting low-level immunity generated by prior exposure to TAAs (18, 46).

**Table 1:** Key inclusion and exclusion criteria.

Inclusion/exclusion criteria	
Major Inclusion Criteria	Participants with genetically confirmed LS with one of the following: MLH1, MSH2/EPCAM and MSH6 genotypes with prior history of ≥1 colorectal neoplasms^1^ OR PMS2 genotype with prior history of colorectal cancer(s) (but no active cancer for 6 months) At least part of the descending/sigmoid colon and/or rectum intact At least 6 months from any cancer-directed treatment (such as surgical resection, chemotherapy, immunotherapy, or radiation) Age ≥ 18 years of age ECOG^2^ performance status ≤ 1 (Karnofsky Performance Status ≥70%) Adequate organ and marrow function
Major Exclusion Criteria	History of organ allograft or other history of immunodeficiency Known human immunodeficiency virus (HIV) with CD4 count < 540, hepatitis B virus (HBV), or hepatitis C virus (HCV) infection^3^ Receiving systemic treatment with corticosteroids (>10mg daily prednisone equivalents) or other immunosuppressive medications within 3 months of vaccination History of allergic reactions attributed to compounds of similar chemical or biologic composition to adenovirus-based vaccines or NAI Pregnancy History of severe side effects or allergic reactions to previous adenovirus-based vaccines (such as Johnson and Johnson COVID vaccine)

^1^ Colorectal neoplasms would include tubular or tubulovillous adenoma(s) or sessile serrated polyps/adenomas/lesion(s) or traditional serrated adenoma(s), and/or colorectal cancer(s) (but no active cancer for 6 months).

^2^ ECOG: Eastern Cooperative Oncology Group.

^3^ Subjects with laboratory evidence of cleared HBV and HCV infection will be eligible.

^4^ NAI, nogapendekin alfa inbakicept.

### 2.4 Vaccines and NAI

#### 2.4.1 Recombinant Tri-Ad5 vaccines

Recombinant Tri-Ad5 vaccines, also known as ETBX-081, are a combination of three investigational adenoviral vaccines (ETBX-011, ETBX-051, and ETBX-061) targeting the TAAs CEA, MUC1, and brachyury, respectively (34). The antigens are encoded as transgenes in proprietary third-generation adenoviral gene delivery vectors containing unique deletions of the viral DNA polymerase and the pre-terminal protein region (Ad5 [E1−, E2b−]) designed to inhibit viral replication and avoid immunological clearance to promote potent immune responses against the targeted tumor antigens in Ad-immune hosts (34–36). To further enhance immunogenicity, the transgenes were designed to include T-cell enhancer agonist epitopes of CEA, brachyury, and MUC1 (25, 30–32) including those of the C-terminus of MUC1 (which has been shown to be an oncogene) (33). Each of the three vaccines that make up ETBX-081 (ETBX-011, ETBX-051, and ETBX-061) was supplied as a sterile, clear solution in a 2 mL single-dose vial at a concentration of 5×10^11^ viral particles per mL of formulation buffer (20 mM Tris, 25 mM NaCl, 2.5% glycerol, pH 8.0). Each vial contained 1 mL of extractable vaccine. The matching placebo was normal saline.

#### 2.4.2 NAI

NAI is a first-in-class immunoglobulin G (IgG) 1-Fc engineered cytokine fusion protein complexed with IL-15 (47–49). NAI is a soluble complex consisting of two protein subunits of a human IL-15 variant (nogapendekin alfa) bound with high affinity to a dimeric human IL-15 receptor alpha (IL-15Rα) sushi domain/human IgG1 Fc fusion protein (inbakicept). NAI is supplied in a 2 mL single dose/use vial containing 0.6 mL of NAI (extractable volume of 0.5 mL) at a concentration of 1 mg/mL or 2 mg/mL. The matching placebo was normal saline.

### 2.5 Randomization/stratification

Participants eligible for the RCT are randomized to either the treatment arm or the control arm in a 1:1 ratio. Randomization is stratified based on the genotype (MLH1/MSH2/MSH6/EPCAM versus PMS2), sex, and remnant length of the colon (rectosigmoid colon versus longer).

### 2.6 Life cycle of the trial for each participant

**Figure 2:**
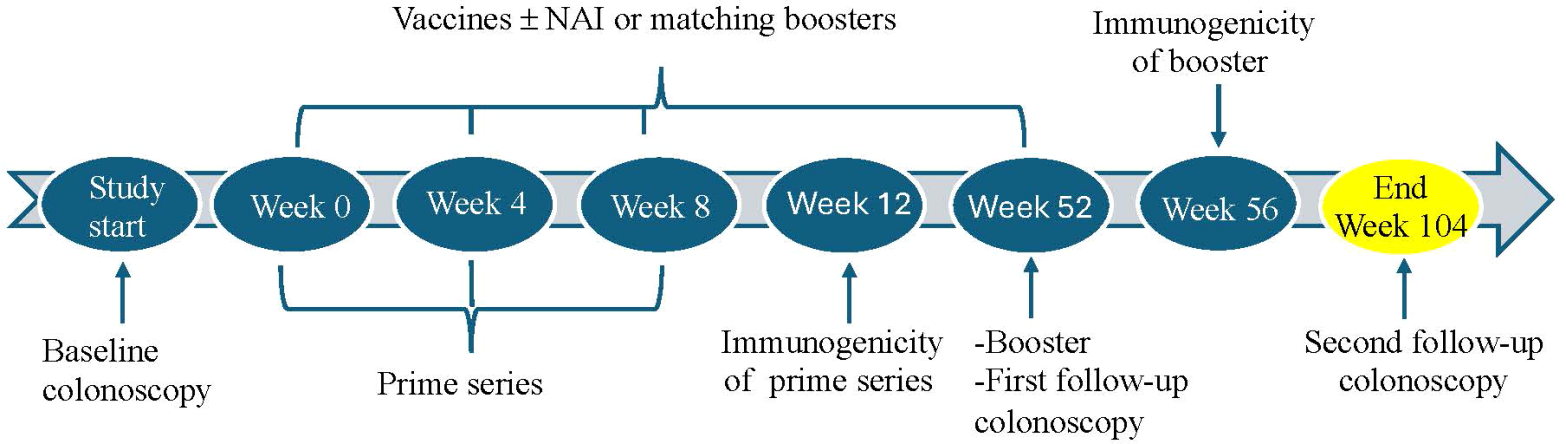
Timeline of study procedures and intervention. NAI, nogapendekin alfa inbakicept.

After pre-screening based on a review of clinical registries and clinic and endoscopic schedules, the participants are approached by the study team. After informed consent is signed, the participants are registered and complete the baseline procedures, including screening labs, colonoscopy, and baseline questionnaires. If they meet eligibility, within 42 days of signing the informed consent, they receive the prime series of SP1 vaccines followed by a booster of the three vaccines at 52 weeks after enrollment. In SP2, they receive the prime series of vaccines along with NAI, followed by a booster series of the three vaccines plus NAI at 52 weeks after enrollment. During the RCT phase, the participants receive either the three vaccines along with NAI or a total of four matching placebo injections. All participants in SP1, SP2, and RCT undergo two follow-up colonoscopies: the first at one year after baseline and the second at two years after baseline, at which time the participant completed study participation. At that time, the participant exits the study. Periodic research blood draws are done throughout the study. There are a total of eight in-person visits as well as three standard-of-care (SOC) colonoscopies for all participants (**Figure 2**), along with telephone calls or email contacts at specified intervals throughout the study.

### 2.7 Study endpoints

#### 2.7.1 Primary endpoint

The primary endpoint of the trial is the cumulative incidence rate of the composite endpoint of adenomas (tubular and tubulovillous), sessile serrated lesions, advanced adenomas, and CRC at two follow-up colonoscopies after intervention. Hyperplastic polyps of any size in the rectosigmoid colon are excluded from the primary endpoint.

#### 2.7.2 Secondary and exploratory endpoints

Secondary endpoints of the RCT include evaluating the safety and tolerability of the Tri-Ad5 vaccines with NAI, the effect of clinical factors such as medication and high-risk behaviors (smoking and alcohol intake) on immune responses, and measuring patient-reported outcomes of vaccine uptake, cancer-specific distress, and quality of life. A key secondary endpoint is understanding the impact of intervention on the incidence of extracolonic malignancies.

Multiple exploratory endpoints are proposed (**Supplementary Table 1**). The key exploratory endpoint is the ability of Tri-Ad5 vaccines in combination with NAI to generate at least a two-fold increase in T-cell responses (cell-mediated immunity) directed against any or all of the TAAs in the vaccine at 12 weeks (early immune response) and at 56 weeks (long-term memory response), measured via intracellular cytokine staining (20, 50). Extensive analysis of soluble analytes will also be performed using the Olink platform (ThermoFisher Scientific Inc.), along with evaluation of peripheral blood immune cell subsets to provide further insights into the immune effects of the intervention (40, 51).

Another important exploratory endpoint is the effect of intervention on the prevalence of MMR-deficient crypts—the purported origin of colorectal cancers in LS (52)—in the colonic mucosa.

### 2.8 Adverse events and safety monitoring

Adverse events (AEs) are coded according to the NCI CTCAE v 5.0. AEs are classified as reactogenic and non-reactogenic. All participants underwent physical exams as is standard for clinical trials. The incidence of skin reactions is discussed in the Results, section 3.4. During SP1 and SP2, continuous safety monitoring was performed by the Safety Committee, which is composed of two study principal investigators, the NCI medical monitor and nurse consultant, and the study statistician. During SP1 and SP2, the cumulative safety data were reviewed weekly and *ad hoc* when a serious adverse event (SAE) was reported. A sequential probability ratio test (with one-sided 0.05 significance level) was initiated after the first three participants were enrolled (53). We defined acceptable toxicity probability (grade ≥ 3) to be ≤0.15 and unacceptable toxicity probability ≥0.30. Subsequently, during the RCT phase, weekly reports of all AEs of grades 3 and higher are reviewed by the Medical Monitor and shared with the principal investigators.

We had planned to halt the RCT if 20% of all participants (once at least 10 participants were accrued) experienced a grade 3 AE, except for the reactogenicity events or if any participant experienced grade 4 or 5 event(s). If this were to happen, we had planned to continue the study with modifications or terminate the study in consultation with the CP-CTNet DSMB, which meets yearly to review the study. It also met after all the participants in SP1 and SP2 had received the third series of injections to make recommendations about study continuation to the RCT phase.

### 2.9 Study procedures

#### 2.9.1 High-quality colonoscopy

After a baseline standard of care (SOC) colonoscopy, each participant undergoes two follow-up SOC colonoscopies at Years 1 and 2 after the baseline, following standardized colonoscopy preparation instructions. The bowel preparation is rated according to the Boston Bowel Preparation Scale (54). Endoscopists use high-definition endoscopes to evaluate the colonic mucosa twice during the same colonoscopy, as tandem studies have shown a miss rate of precancers on a single withdrawal to be as high as 50% (55–58). Polyp morphology is classified according to the Paris classification (59). Colonoscopies and polypectomies are carried out according to the guidelines of the American Society of Gastrointestinal Endoscopy (60). Polyps are removed as SOC and collected in formalin. In addition, research biopsies are being obtained per protocol.

#### 2.9.2 Participant questionnaires

All participants will complete the following questionnaires: tobacco (61) and alcohol use (adapted from the 2013 National Health and Nutrition Examination Survey (NHANES) alcohol use survey), cancer-specific distress (62, 63), health-related quality of life (64), pros and cons of participation (adapted from (65, 66)), past vaccination history, and the end of the study “was it worth it?” (67). Potential participants who declined the study were asked to complete a short survey about “reasons for declining participation”.

### 2.10 Laboratory procedures

Antigen-specific T cells will be determined by intracellular cytokine staining, as has been done in other studies (20, 50). To interpret the results, a patient will be scored as developing a response to a TAA if a) a patient has more than 250 CD4^+^ or CD8^+^ T cells that produced IFN-γ, TNF-α, or IL-2 or were positive for CD107a per 1 × 10^6^ cells that were plated at the start of the assay and b) an increase greater than two-fold post-therapy vs pre-therapy. Multifunctional T cells, defined as CD4^+^ or CD8^+^ T cells expressing two or more of IFN-γ, TNF-α, IL-2, or CD107a, will also be assessed (20, 50). Antibody responses will be measured by standardized established ELISA assays as previously published (68).

As part of pre-trial planning, we stained LS-associated cancers and precancers for the three TAAs (see Results below). IHC was performed for preliminary data to detect MUC1 (clone Ma552, Novocastra, 1:100 dilution) and CEA (clone II-7, Dako, 1:100 dilution); immunofluorescence was used to detect brachyury (clone 54, Abcam, 1:1000 dilution) (69) along with appropriate controls. Immunohistochemical expression was assessed for staining intensity (0= no expression, 1 = weak intensity, 2 = moderate intensity, 3 = strong intensity), if seen in at least 10% of the cells.

TAA staining will be performed during the RCT phase in colorectal neoplasms from study participants pre- and post-vaccination using normal colonic mucosa for comparative analysis, as previously described (70–72). RNAseq will be performed on biopsies of normal colonic mucosa and colorectal neoplasms to evaluate changes in the immune profile and the abundance of resident immune cell types after intervention. Serum soluble factors and cytokines, and immune cell subsets in peripheral blood will be measured using standardized assays.

### 2.11 Statistical considerations

#### 2.11.1 Sample size calculations

The planned sample size for the two safety phases was 20, with 10 participants for each phase. This sample size was chosen to allow estimation of AE rates with adequate precision levels. Specifically, with the planned sample sizes, AE rates for each safety phase cohort were estimated with a standard error of less than 20%. For the RCT phase, the null hypothesis that the cumulative event probability for the primary endpoint in the control arm is less than or equal to the cumulative event probability in the investigational arm will be tested against the cumulative event probability in the control arm being greater than in the investigational arm. Based on the results from prior non-intervention trials in which tandem colonoscopies were performed (55–58), it was estimated that the cumulative event rate in the control arm was 40%. With the proposed intervention, a reduction in the cumulative event rate to 20% was anticipated. A sample size of 46 participants per arm is required to detect the anticipated difference in the cumulative event rates between arms with 80% power at the one-sided 0.1 significance level. Accounting for a screen failure rate of up to 15% and an attrition rate of up to 33%, the planned total sample size for the safety phases and RCT phase was 186 participants.

#### 2.11.2 Statistical analysis plan

Since this is an early-phase efficacy trial designed to detect a preventive efficacy signal of the proposed vaccine combination, the modified intent-to-treat (mITT) population will be used as the primary analysis population for the efficacy analysis and the evaluations of immunological and clinical endpoints. The mITT population will consist of all eligible participants who are randomized and complete the two follow-up colonoscopies. The primary endpoint will be compared between the two study arms in the RCT using the stratified Mantel-Haenszel test, in which the randomization stratification factors will be included as strata. Secondary endpoints will be compared between the study arms using a Wilcoxon rank sum test. Patient-reported outcomes will be analyzed descriptively. Safety endpoints will be analyzed using the safety population, defined as all study participants who received at least one dose of study treatment. AEs will be analyzed descriptively in tabular format, stratified by type, attribution, and severity. Comparisons of AE rates between study arms during the RCT phase will be conducted using a chi-square test or Fisher’s exact test, as appropriate.

#### 2.11.3 Interim analysis

Although an interim analysis for futility was initially planned for the RCT phase, the rapid accrual rate suggested that accrual was likely to finish before the participants reached the timing of the interim analysis (which was based on the findings at the time of the first follow-up colonoscopy). Consequently, the protocol was amended and the interim analysis for futility was removed.

## 3 Results

### 3.1 Expression of TAAs in Lynch precancers and cancers

**Figure 3:**
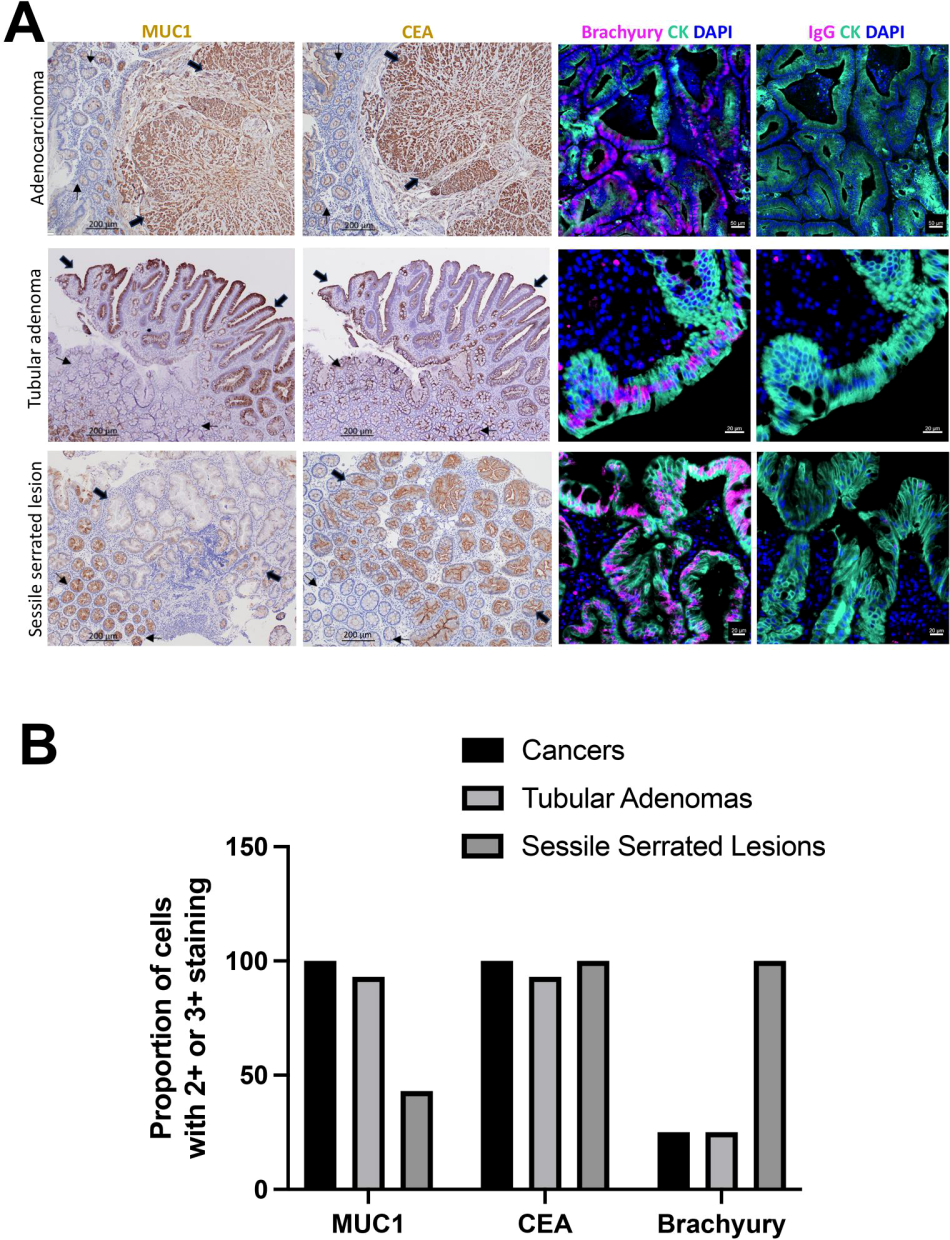
TAA staining and quantification in Lynch cancers and pre-cancers. In **(A)**, left 2 columns show IHC for MUC1 (cytoplasmic, 1:400) and CEA (cytoplasmic/membranous, 1:250). Note higher expression of both in the cancer and of MUC1 in the tubular adenoma (thick arrows) compared to normal epithelium (thin arrows). The sessile serrated lesion (thick arrows) shows reduced MUC1 and increased CEA compared to normal epithelium (thin arrows). Original magnification X100; scale bar: 200 μm. Right two columns show immunofluorescence for brachyury (pink), CK (cytokeratin, green), DAPI (blue); IgG staining was negative in all tissues (rightmost panels). Scale bars: 20-50 μm, as indicated. In **(B)**, TAA staining is quantified across various histologies.

Expression of the three TAAs (MUC1, CEA and brachyury) was examined in a limited number of available samples but these confirmed the expression of various TAAs in both precancers and cancers, albeit with differential expression across histology. We included 13 LS-related CRC and 22 precancers (15 tubular adenomas and 7 sessile serrated lesions) (**Figure 3**). Overall, MUC1 expression was higher in tubular adenomas whereas CEA and brachyury expression were higher in sessile serrated lesions (**Figure 3B**). Among CRC, the MUC1 expression was higher (69% 3+, 31% 2+) than that in the normal mucosa (7.6% 3+, 92.4% 2+) and the CEA expression was higher (92.3% 3+, 7.7% 2+) than that in the normal mucosa (15.3% 3+, 84.7% 2+). Among tubular adenomas, the MUC1 expression was higher (60% 3+, 33.3% 2+) than that in the normal mucosa (100% 2+) and the CEA expression was higher (86.6% 3+, 6.6% 2+) than that in the normal mucosa (6.6% 3+, 93.3% 2+). Among sessile serrated lesions, the MUC1 expression was lower (0% 3+, 42.9% 2+) than that in the normal mucosa (14.2% 3+, 85.7% 2+) and the CEA expression was higher (100% 3+) than that in the normal mucosa (28.6% 3+, 71.4% 2+). Brachyury expression was observed by immunofluorescence (IF) in 25% of CRC, 25% of tubular adenomas and 100% of sessile serrated lesions.

### 3.2 Enrollment and demographics

**Figure 4:**
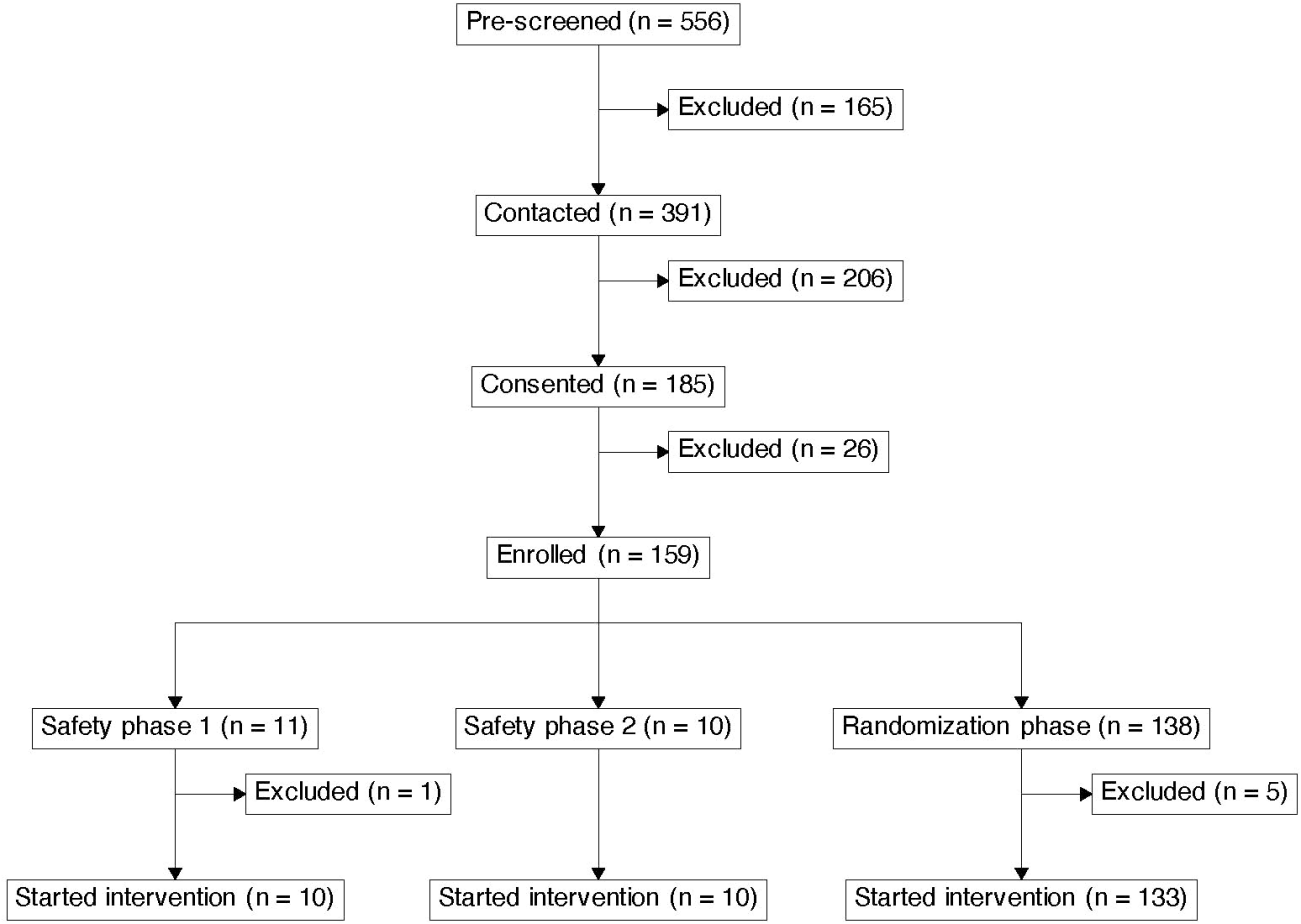
Consort diagram.

Although the accrual to both safety phases and the RCT is complete (**Figure 4**), the study is ongoing and the final results will not be available until all participants complete their year 2 colonoscopy. Therefore, we present here only limited data from SP1 and SP2.

Enrollment to SP1 and SP2 occurred sequentially. Sixty potential participants were pre-screened, of these 24 were consented and completed screening, and 21 participants were enrolled. One participant was enrolled to SP2 but did not start intervention due to COVID infection. Participant demographics are shown in **Table 2**. Twenty participants (median age 57.5 [range 42–75], 70% female, 15% minorities) completed intervention, including the initial three cycles of treatment, 12-week follow-up, and Week 52 vaccination. All 10 SP1 participants and five SP2 participants have completed their Week 104 colonoscopy (study exit for these participants); the other five SP2 participants have not yet reached week 104.

**Table 2:** Patient demographics from SP1 and SP2.

Variable	Safety Phase 1 N=11	Safety Phase 2 N=10
**Age (median, range)**	58 (42-73)	55 (42-75)
**Gender, n (%)**		
Female	8 (73%)	7 (70%)
Male	3 (27%)	3 (30%)
**Ethnicity, n (%)**		
Not Hispanic or Latino	11 (100%)	10 (100%)
**Race, n (%)**		
Asian	0 (0%)	1 (10%)
Black	0 (0%)	1 (10%)
More than one race	0 (0%)	1 (10%)
White	11 (100%)	7 (70%)
Unknown or not reported	0 (0%)	0 (0%)

**Figure 5:**
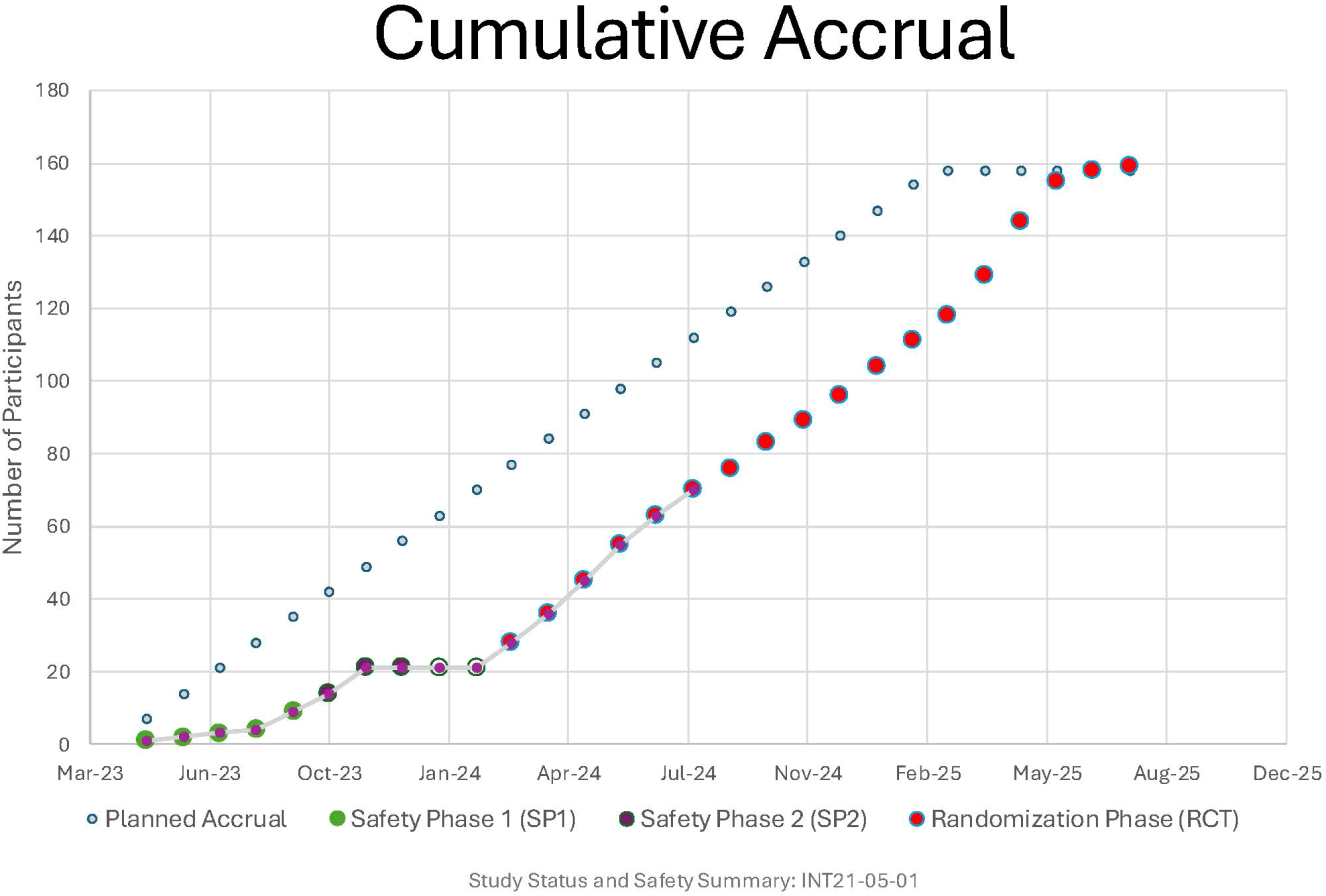
Accrual quality improvement program (AQuIP) report. Light blue circles represent planned accrual while non-blue (green, purple, and red) circles represent actual accrual.

Although the rate of accrual was initially slow with only a few sites, the rate accelerated substantially as more sites opened. We monitored accrual closely using the NCI’s Accrual Quality Improvement Program (**Figure 5**) (73). Even with the initial slower pace, after all the sites were activated, the slope of actual accrual closely mimicked that of the planned accrual (**Figure 5**). Accrual was halted when all 10 participants started intervention in SP2 and did not begin to the RCT until all SP2 participants reached the Week 12 safety assessment. The DSMB reviewed the safety data and approved re-opening of accrual.

### 3.3 Adverse events

**Figure 6:**
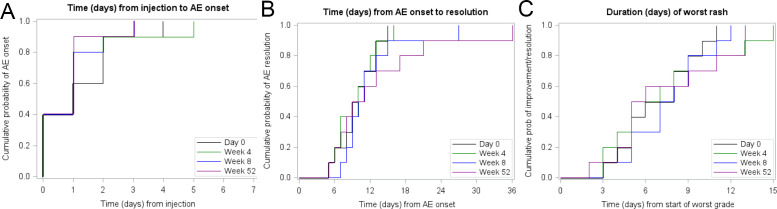
Time course of NAI-induced skin reactions. Data are from participants in Safety Phase 2 over four different time points.

All AEs occurring in 20% or more participants in either SP1 or SP2 are shown in **Table 3**. Ten participants in SP1 reported 139 AEs and 10 participants in SP2 reported 178 AEs. The most common treatment-related AEs were expected reactogenic events. Skin reactions were graded as vaccination complications per FDA Guidance for Industry (Sept. 2007); skin reactions >10 cm were considered grade 3. The onset of skin reaction was within one day of the NAI injection with a total duration of approximately 10 days. No participant had skin breakdown, necrosis or interference with self-care activities. In all cases, the skin reaction either resolved completely or was downgraded to grade 1 or 2 between the injection cycles. There was no difference in the timing or duration of the skin reaction between the injection cycles (**Figure 6**). Other common AEs (attributed as possible, probable, and definite; >20% frequency) during SP1 and SP2 are listed in **Table 3**. AEs were predominantly grade 1. No participants discontinued intervention during either phase. All non-reactogenic AEs completely resolved between treatment cycles and all participants received the next treatment on schedule. Two SAEs were reported, both in SP1, as self-limiting vaginal hemorrhage after hysterectomy and sleep apnea with both considered unrelated to study treatment. The safety profile was reviewed by the DSMB and the trial was recommended for continuation to the RCT phase, which has recently completed accrual but is otherwise ongoing.

**Table 3:** AEs in SP1 and SP2.

Overall summary of AEs by worst grade per participant with attribution of at least possibly treatment related (CTCAE v. 5.0)		
AE grade	Safety phase 1 (N=10)	Safety phase 2 (N=10)
**Grade 1**	3 (30%)	0 (0%)
**Grade 2**	5 (50%)	0 (0%)
**Grade 3**	2 (20%)^1^	10 (100%)^1^
Detailed AE reporting^2^		
**CTCAE Term**	**SP1**	**SP2**
Anorexia		
*Grade 1*	1 (10%)	2 (20%)
*Grade 2*	0%	0%
Arthralgias		
*Grade 1*	1 (10%)	5 (50%)
*Grade 2*	0%	0%
Chills		
*Grade 1*	6 (60%)	7 (70%)
*Grade 2*	0%	1 (10%)
Diarrhea		
*Grade 1*	2 (20%)	3 (30%)
*Grade 2*	0%	0%
Fatigue		
*Grade 1*	7 (70%)	8 (80%)
*Grade 2*	0%	0%
Fever		
*Grade 1*	5 (50%)	9 (90%)
*Grade 2*	1 (10%)	0%
Headaches		
*Grade 1*	6 (60%)	7 (70%)
*Grade 2*	2 (20%)	0%
Hyperhidrosis		
*Grade 1*	0%	2 (20%)
*Grade 2*	0%	0%
Insomnia		
*Grade 1*	2 (20%)	1 (10%)
*Grade 2*	0%	0%
Myalgia		
*Grade 1*	7 (70%)	6 (60%)
*Grade 2*	1 (10%)	0%
Nausea		
*Grade 1*	4 (40%)	4 (40%)
*Grade 2*	0%	0%
Vomiting		
*Grade 1*	0%	4 (40%)
*Grade 2*	1 (10%)	0%
Vaccination complication		
*Grade 1*	1 (10%)	0%
*Grade 2*	6 (60%)	0%
*Grade 3*	3 (30%)	10 (100%)

^1^ All grade 3 AEs were injection site reactogenic events graded as vaccination complication.

^2^ Only AEs with a frequency of 20% or higher in any group were included.

## 4 Discussion

Our trial has been highly successful with regard to accrual for several reasons. The network structure allowed multiple groups to join in via a centralized data management infrastructure. The patient cohort is highly motivated and well informed via shared family experiences and patient advocacy organizations. The study investigators are motivated physicians who take care of these patients in their SOC practices. This first cross-network trial that engaged all the CP-CTNet cancer prevention consortia (each with its individual participating sites) will serve as a holistic model for future cancer interception trials such that early-phase clinical trials can be completed efficiently.

Anti-cancer immunotherapy is powerful. In the Neoadjuvant Immune Checkpoint Inhibition and Novel IO Combinations in Early-Stage Colon Cancer 2 (NICHE2) trial (74), use of neoadjuvant immunotherapy to block two immune checkpoint molecules (programmed cell death protein 1[PD-1] and cytotoxic T-lymphocyte-associated protein 4 [CTLA-4]) in 115 patients (31 with LS) with localized MMR-deficient CRC led to a pathological complete response in approximately two-thirds of patients (74). The fact that harnessing the immune system could eradicate even established tumors in the context of immune tolerance raises the possibility that immune interception applied at the pre-invasive stage (when there is less immunosuppression) could be an effective strategy to eliminate precancerous cells (Schreiber hypothesis) (75). Lynch syndrome is an ideal model for cancer immune-interception because of heightened immune activity (76, 77). However, checkpoint inhibitor therapy may not meet the risk–benefit ratio for prevention; also, published data suggest that ICI therapy alone may not be effective at preventing precancers (78).

The only currently established preventive approach in LS is aspirin (10, 11), although uptake has been limited due to a variety of reasons including perceived risks of high-dose aspirin (12–14). Newer understanding of the effects of aspirin on activating CD8^+^ T cells by blocking platelet production of thromboxane receptor A2 (TXA2) (79) provides novel insights into the efficacy of aspirin in preventing colorectal cancers in the Cancer Prevention Programme 2 (CAPP2) trial (10). The results of the CAPP3 trial that compared low-dose against high-dose aspirin are eagerly awaited.

Vaccines could be a practical, well-tolerated and cost-effective way to activate the immune system for cancer interception in patients with LS. Short-term treatment could lead to long-term prevention, which could help patient uptake. Early-phase trials of vaccine-based secondary prevention in patients with pancreatic cancer with low disease burden have shown a lot of promise (80, 81). Several studies have also demonstrated that various immunomodulatory agents, when used in combinations, can elicit patient-specific tumor neoepitope T-cell responses (82). Recently a vaccine was approved by the FDA for recurrent respiratory papillomatosis in patients with low disease volume following debulking surgery (83).

It should be noted that the adenoviral vaccine platform selected for use in this study has been specifically engineered to contain deletions of both the viral DNA polymerase and the pre-terminal protein region (Ad5 [E1−, E2b−]) with the goal of inhibiting viral replication and avoiding immunological clearance in hosts with pre-existing Ad-immunity (34–36). Of note, this adenoviral vaccine platform has been utilized in multiple clinical trials of patients with advanced cancer, where boosting of T-cell responses against the tumor antigens encoded by the vaccine were noted with repeated vaccinations, and despite the presence of Ad5-neutralizing antibodies (34–36).

The first ever RCT of colorectal cancer immunoprevention using a MUC1 peptide vaccine was successfully completed in a non-Lynch high-risk population that included individuals with advanced precancers (84). The primary endpoint of reduced incidence of colorectal precancers between the intervention and the control group was not shown to be statistically significant; however, in a subgroup analysis, when immune responders (participants who had an immune response at Week 12 and the booster injection) were compared to non-immune responders, MUC1 peptide vaccine reduced the incidence of new precancers by 38% compared with placebo (adjusted RR, 0.41; 95% CI, 0.15–1.11; *P* = 0.08). The immunogenicity rate in the MUC1 peptide vaccine RCT was approximately 40% lower than that seen in the pilot trial (68), emphasizing the importance of quality immune responses on clinical effects. These results informed our current study, leading to the decision to use a combination of vaccines against three cancer antigens (to prevent tumor escape) and the addition of the IL-15 receptor superagonist as an adjuvant to boost immune responses, as discussed earlier. A small number of samples were available to examine the TAA expression. The three TAAs were differentially expressed between the two types of precancers. Among tubular adenomas, expression of both MUC1 and CEA was higher compared with the normal mucosa whereas among sessile serrated lesions, MUC1 expression was lower and CEA expression was higher compared with the normal mucosa. Brachyury was expressed in one-fourth of tubular adenomas but in all SSL sessile serrated lesions. These results support the use of vaccine combinations for cancer interception as individual vaccines may be more effective against a subtype of precancer. In our trial, we will be able to correlate immune responses to individual TAAs and their effect on the incidence of specific pre-cancers.

The ideal clinical endpoint for an intervention trial in individuals with LS would be incident cancer(s), although even in this high-risk population, cancer as an endpoint would have inflated the trial to an impractical size and duration. Even though there is controversy around precursor lesions of colorectal cancers in LS — MMR-deficient crypts (52) versus the conventional adenoma-carcinoma sequence (4) — long-term follow-up studies support the importance of visible precancers during carcinogenesis in LS because colonoscopy can prevent colorectal cancer by allowing removal of precancers (85, 86). Furthermore, unlike MMR-deficient crypts, adenomas and sessile serrated lesions can be identified and quantified during a colonoscopy.

Overall, the participants in the current trial have tolerated the vaccines well. We will have detailed data on the RCT at the conclusion of our study, but based on the experience in SP1 and SP2, vaccine- and adjuvant-based AEs were self-limited. A prominent AE that has been frequently seen in SP2 and the RCT is a large skin reaction at the intra-abdominal injection site of the IL-15 receptor superagonist. This has not been associated with skin breakdown or necrosis and has resolved completely. Additionally, these skin reactions were time-limited and did not interfere with activities of daily living. They were considered manageable in the majority of participants. The attribution as ‘grade 3’ is a bit of a misnomer and is based on FDA guidelines for vaccine development. If we had used the CTCAE v5.0 term of ‘injection site reaction’ instead of ‘vaccination complication’, these skin reactions would have been grade 2. Improved understanding of the time course and behavior of skin reactions during SP2 along with participant and staff education is helping us in retaining participants during the RCT. There were no participant drop-outs during SP1 and SP2; all 20 participants completed the entire vaccine prime and booster series.

To summarize, cancer vaccines have garnered interest as a novel modality for the interception of carcinogenesis and for improving overall outcomes in at-risk individuals or patients with cancer. By combining multiple vaccines with a powerful adjuvant, this trial aims to explore a novel avenue for cancer prevention — CRC and potentially for extracolonic cancers — in individuals with LS. This trial will add to our current knowledge of cancer-immune biology and determine the viability of multitargeted TAA-based cancer vaccines in the prevention setting. Currently, the trial is in its RCT phase, and the results are eagerly awaited.

## Data Availability

The datasets presented in this study can be found in online repositories. The names of the repository/repositories and accession number(s) can be found in the article/**Supplementary Material**.
