# Supplementary material for: Design and preliminary report of a randomized phase IIb clinical trial of multitargeted recombinant adenovirus 5 vaccines against CEA, MUC1, and brachyury (Tri-Ad5) and the IL-15 receptor superagonist nogapendekin alfa inbakicept in Lynch syndrome (TRIAD5-Plus): the first cross-network trial of the Cancer Prevention Clinical Trials Network (CP-CTNet)
Source: Front Immunol. 2026 May 19;17:1809281. doi: 10.3389/fimmu.2026.1809281 (PMC13226594; doi:10.3389/fimmu.2026.1809281)
Supplement: Supplementary file 1 [file Table1.docx]

| **Supplementary Table 1: Exploratory endpoints** |
| --- |
| - To determine the ability of the Tri-Ad5 vaccines+N-803 to generate a 2-fold increase in T cell responses (cell-mediated immunity) at Week 12 (early immune response) and at Week 56 (long-term memory response) - To evaluate circulating anti-MUC1 IgG (antibody-mediated immunity) after Tri-Ad5 vaccines+N-803 - To compare the expression of the three tumor associated antigen (TAAs): MUC1, CEA and brachyury in colorectal neoplasms before and after Tri-Ad5 vaccines+N-803 - To evaluate changes in the immune profile and abundance of resident immune cell types in colonic mucosa after vaccination with Tri-Ad5 vaccines+N-803 using mRNAseq and IHC - To test the effects of the vaccines alone or in combination with N-803 on specific immune subsets of PBMCs and serum soluble factors and cytokines. - To compare the expression of stem cell markers in colorectal neoplasms before and after Tri-Ad5 vaccines+N-803 - To compare the number of mismatch repair deficient (MMR-deficient) crypts at baseline to the number of MMR-deficient crypts at the one-year post-vaccination colonoscopy both overall and on a per-patient basis |
